# Active mode locking of quantum cascade lasers in an external ring cavity

**DOI:** 10.1038/ncomms11440

**Published:** 2016-05-05

**Authors:** D. G. Revin, M. Hemingway, Y. Wang, J. W. Cockburn, A. Belyanin

**Affiliations:** 1 Department of Physics and Astronomy, The University of Sheffield, Sheffield S3 7RH, UK; 2 Department of Physics and Astronomy, Texas A&M University, College Station, Texas 77843, USA

## Abstract

Stable ultrashort light pulses and frequency combs generated by mode-locked lasers have many important applications including high-resolution spectroscopy, fast chemical detection and identification, studies of ultrafast processes, and laser metrology. While compact mode-locked lasers emitting in the visible and near infrared range have revolutionized photonic technologies, the systems operating in the mid-infrared range where most gases have their strong absorption lines, are bulky and expensive and rely on nonlinear frequency down-conversion. Quantum cascade lasers are the most powerful and versatile compact light sources in the mid-infrared range, yet achieving their mode-locked operation remains a challenge, despite dedicated effort. Here we report the demonstration of active mode locking of an external-cavity quantum cascade laser. The laser operates in the mode-locked regime at room temperature and over the full dynamic range of injection currents.

## Abstract (web summary)

Compact, integrated mode-locked lasers can produce ultrashort pulses of light in the visible and near infrared, but are more difficult to achieve in the mid-infrared. Here, the authors demonstrate active mode locking of an external-cavity quantum cascade laser emitting at 5.2 micrometres at room temperature.

## Graphical abstract

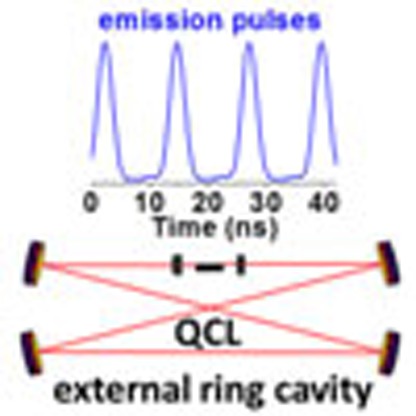

Quantum cascade lasers (QCLs) are based on electron intersubband transitions in coupled quantum wells and superlattices; they offer unique flexibility in engineering the emission wavelength and the width of the gain spectrum. Recently, free-running QCLs with octave-spanning lasing spectra^1^ and a long-lived phase coherence of laser modes^2^,^3^ were demonstrated and promising applications such as dual-comb spectroscopy were identified^4^. However, attempts to mode-lock mid-infrared QCLs have had limited success. Passive mode locking has been found to be impossible in QCLs. The reason lies in the fundamental difference between the dynamics of QCLs and virtually every other kind of laser. For the intersubband transitions, the ultrafast carrier relaxation time limits the gain recovery time to just a few picoseconds^5^. This is much shorter than the round-trip time of 40–70 ps for a mid-infrared QCL with a typical cavity length of a few mm. Under these conditions the tails of the pulse experience unsaturated gain and therefore amplification, thus preventing the formation of stable mode-locked pulses.

Active mode locking (AML) by gain modulation at the period equal to the round-trip time was identified as the only viable route to QCL mode locking^6^,^7^,^8^,^9^. In this case a stable pulsed regime is achieved by periodically opening a window of net gain on a timescale that is short compared with the cavity round-trip time. In terahertz QCLs this could be achieved by applying microwave modulation to laser chips^10^. In mid-infrared QCLs, the only successful demonstration of AML was achieved in two-section monolithic cavity lasers with a so-called ‘super-diagonal' design with a significantly longer gain relaxation time, at the expense of overall device performance. The AML was observed only close to the threshold and only at cryogenic temperatures^6^.

A more robust and potentially more practical approach to AML of QCLs has been proposed (but not achieved until the present publication) for lasers operating in free-space external cavities^9^,^11^. In this case, one can utilize a high-performance QCL, electrically modulate the whole laser chip, thus achieving deep gain modulation, while at the same time maintaining a very short gain window when compared with the round-trip time of the light along the external cavity. This extends the dynamic range of currents where the AML can be observed far above threshold. In addition, a long external cavity has round-trip frequencies reduced from tens of gigahertz in monolithic QCLs to hundreds of megahertz, providing greater control for gain or loss modulation by using low-cost instrumentation and makes it easy to incorporate additional optical elements or a gas cell inside the cavity.

In the following we demonstrate the AML of a QCL operating in an external ring cavity at room temperature. This is the demonstration of room-temperature mode locking in QCLs of any kind. Our results show that, contrary to widespread belief, neither short (of the order of 1 ps) gain recovery time nor the spatial hole burning (SHB) in the gain medium prohibits AML of QCLs. The observed AML regime is very robust: the emission pulses maintain strict periodicity for at least 10^8^ round trips and they persist over the full dynamic range of the injection currents (from the laser threshold and up to the laser roll-over), for all tested cavity lengths, and at the fundamental and its harmonics modulation frequencies of ∼80–400 MHz.

## Results

### Pulsed operation under resonant modulation

**Figure 1:**
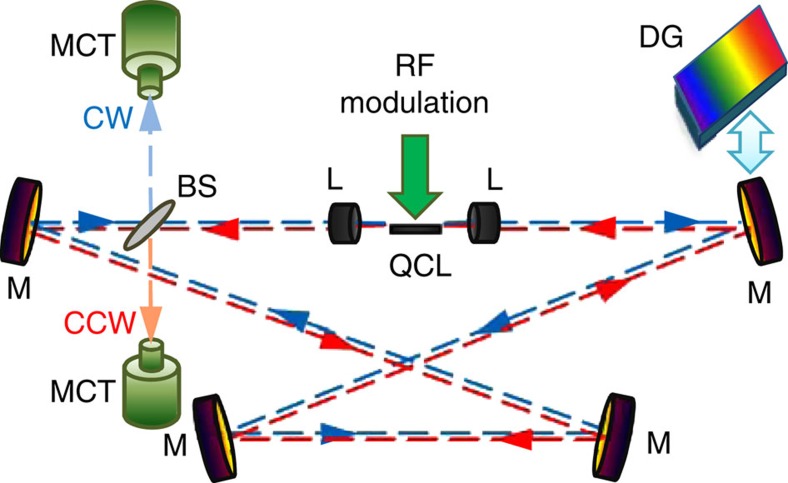
Optical set-up of free-space external ring cavity quantum cascade laser. BS, beam splitter; CCW, counter clockwise direction; CW, clockwise direction; DG, diffraction grating; L, aspheric lenses; M, mirrors; MCT, detector; QCL, quantum cascade laser.

**Figure 2:**
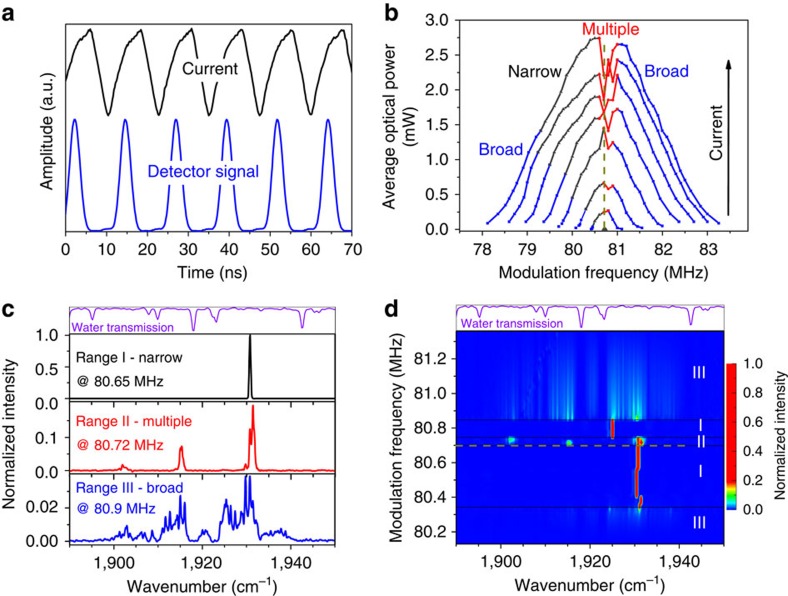
Performance of external ring cavity QCL. (**a**) Periodic emission pulses and driving current for the modulation near the round-trip frequency of 80.7 MHz. The current signal is vertically shifted for clarity. (**b**) Average out-coupled optical power for the QCL driven near the resonant frequency with the current amplitude from 1.01·*I*_th_ to 1.7·*I*_th_ and the incremental step of 0.1·*I*_th_ from curve to curve, where *I*_th_ is the threshold current. Dashed vertical line indicates the position of the resonance frequency. (**c**) Typical emission spectra: narrow peak, multiple peak and broad. Transmission spectrum for water in the air is shown as a reference. (**d**) Detailed emission spectra for the QCL driven near the resonant frequency with the current amplitude of 1.15·*I*_th_. Dashed horizontal line indicates the position of the resonance frequency. Compressed rainbow colour map is used to enhance the visibility of the low-intensity broad spectra compared with the strong narrow peak ones. The spectra presentred in **c** and **d** are normalized to the highest intensity of the narrow peak emission in range I.

A 4.5-mm-long, 9-μm-wide *λ*∼5.25 μm buried heterostructure QCL (ref. 12), with both facets having anti-reflection coatings is used as a gain medium and driven with a current of variable frequency and amplitude. The optical and electrical arrangements of the free-space external ring cavity set-up (Fig. 1) are described in more details in Methods section. When the laser chip is pumped with the current periodically modulated near the round-trip frequency of ∼80.7 MHz for the ∼3.8-m-long cavity or its higher harmonics of up to ∼403 MHz, the ring cavity QCL emits a periodic sequence of pulses with the repetition rate equal to the frequency of the driving current (Fig. 2a). The emission pulses are well separated and have a stable amplitude. The observed shape and duration of these pulses are mainly defined by the relatively long ∼2-ns rise time of the detector and to some extent by the bandwidth of the oscilloscope. We believe that the actual duration of these pulses is likely to be much shorter, as discussed below.

The average output power of the external ring cavity QCL driven with the current modulated at frequency near the round-trip frequency and the amplitude from 1.01·*I*_th_ to 1.7·*I*_th_ is presented in Fig. 2b, where *I*_th_ is the laser threshold current. The round-trip frequency initially estimated from the length of the ring cavity is identified more precisely in experiment by establishing the repetition rate of the driving current for which the laser emission is observed at the lowest current. If the modulation frequency is detuned from the resonance frequency, the pulses are still generated but at higher current values. It was found that the laser emits in both clockwise (CW) and counter CW (CCW) directions with identical characteristics but different intensities. The emission in the CCW direction is ∼70% of the optical power for CW direction. We explain this asymmetry by slightly different reflectivities of the anti-reflection coatings at the laser chip facets. The experimental data presented here are for the CW direction only. The existence of the waves propagating in both CW and CCW directions means that a standing wave pattern can be formed inside the QCL chip, consequently leading to the formation of a transient population grating, or SHB. This effect has been shown to destroy mode locking in monolithic QCLs (refs 13, 14) since scattering on the population grating leads to proliferation of modes with uncorrelated phases. Our experimental results indicate that the AML of external-cavity QCLs is possible even in the presence of SHB.

The maximum average output optical power out-coupled from the cavity using the beam-splitter increases linearly with the current and is almost 3 mW for the current amplitude of 1.7·*I*_th_ (Fig. 2b). Beyond this point the emission intensity stops increasing, indicating that light-current roll-over conditions have been reached, in good agreement with the laser performance in the continuous wave or pulsed, non-resonant regimes. The frequency detuning range in which the laser emits pulses is the narrowest at threshold but increases rapidly for higher driving currents spreading ∼3% both above and below the round-trip frequency. As expected, the output optical power reaches its maximum at the modulation frequency close to the round-trip frequency for all values of current and rapidly decreases with detuning, when the window of net gain opens at times slightly out of phase with the round-trip time of the laser light.

### Laser spectra under resonant modulation

The emission spectra of the laser change dramatically as modulation frequency is detuned from the resonance (Fig. 2c). Slightly below the round-trip frequency (the data marked in black colour in Fig. 2b) the emission spectra consist of a very narrow, resolution limited, single peak, with the full width at half maximum (FWHM) of ∼0.45 cm^−1^ (∼13.5 GHz), containing up to 170 longitudinal modes of the ring cavity resonator. At the resonance frequency and slightly above, the emission becomes much less stable with the spectra often having several peaks with energy positions and intensities which abruptly jump/shift with slight variation in the modulation frequency or optical alignment (the data marked in red colour in Fig. 2b). With further detuning from resonance, both above and below (the data marked in blue colour), the emission spectra abruptly become very broad, with FWHM of up to 30 cm^−1^ (∼900 GHz), and containing more than 11,000 modes of the ring cavity. These broad spectra have relatively uniform shapes with only dips corresponding to the water vapour absorption lines. The detailed dependence of the spectra on the modulation frequency is illustrated in Fig. 2d for the current amplitude of 1.15·*I*_th_. The frequency range areas marked as I, II and III correspond to the narrow single peak, multi-peak emission and the broad spectra, respectively. The origin of the narrow spectra in the vicinity of the resonance and the broadening of the spectra with modulation frequency detuning are discussed in more details in the theoretical modelling part.

Very similar dependence on the frequency detuning and, more importantly, the existence of the same types of the emission spectra are present if the driving current is modulated at the second and the third harmonics of the round-trip frequency (∼161 and 242 MHz). Emission has also been detected for the modulation at the fourth and the fifth harmonics (∼323 and 403 MHz). However, the operation of the interface driving board becomes much less efficient at higher frequencies. AML at higher harmonics yields pulses at higher repetition rates for any given cavity length.

The maximum out-coupled peak optical power is estimated to be ∼12 mW if the emission pulses are indeed 3–4 ns long. However, it is likely that this duration is limited by the resolution of the detector, since the same pulses are observed for all types of the emission spectra, both narrow and broad ones. The width of the observed narrow peak spectra under modulation below the round-trip frequency is 0.45 cm^−1^, which should correspond to pulses of 75 ps duration and with a peak power of several hundred mW, assuming perfect mode locking. To confirm the pulse duration, one would have to use an ultrafast quantum well infrared photodetector or interferometry autocorrelation technique. Such measurements will be the subject of future research.

### Tuning of laser spectra with a diffraction grating

**Figure 3:**
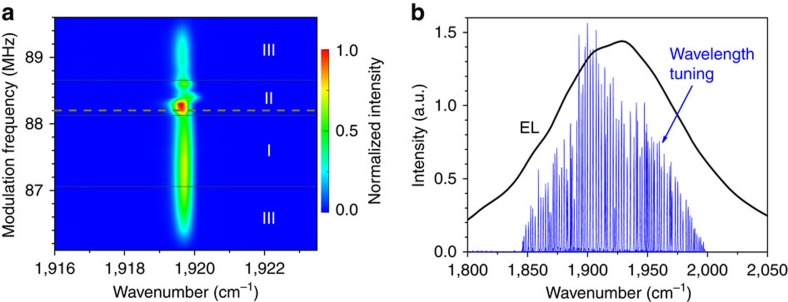
Emission spectra with a diffraction grating. (**a**) The QCL is modulated near the resonant frequency of 88.2 MHz and driven with the current amplitude of 1.15·*I*_th_, where *I*_th_ is the threshold current. The angle of the diffraction grating is fixed. The frequency ranges I, II and III correspond to different shapes of the radio frequency intermode beat spectra (Fig. 4a). Dashed horizontal line indicates the position of the resonance frequency. (**b**) Set of the narrow single-peak spectra with full-width half maximum of∼0.45 cm^−1^ at the current amplitude of 1.6·*I*_th_ demonstrating the wavelength tuning by rotating the diffraction grating (for the modulation frequencies in range I). For comparison: electroluminescence (EL) measured with 20 cm^−1^ resolution.

To control the central emission wavelength of the mode-locked ring cavity QCL for potential applications in spectroscopy, one of the external flat mirrors in the ring cavity (Fig. 1) was replaced by a 300 lines per mm diffraction grating. The resulting length of the aligned cavity turned out to be a bit shorter: ∼3.4 m, with the round-trip frequency of ∼88.2 MHz. The use of the grating strongly limits the emission wavelength range of the mode-locked QCL. The QCL is forced to operate only near the wavelength defined by the incident angle of the diffraction grating for all modulation frequencies near resonance (Fig. 3a). Very narrow spectra of FWHM∼0.45 cm^−1^ (∼13.5 GHz) are observed for all detuning frequencies. The rotation of the diffraction grating leads to the emission wavelength tuning in the range of ∼150 cm^−1^ (Fig. 3b), very close to the FWHM of ∼160 cm^−1^ of the electroluminescence spectrum, measured for this anti-reflection coated QCL under non-resonant pulsed pumping without feedback from external mirrors.

### Radio frequency spectra

**Figure 4:**
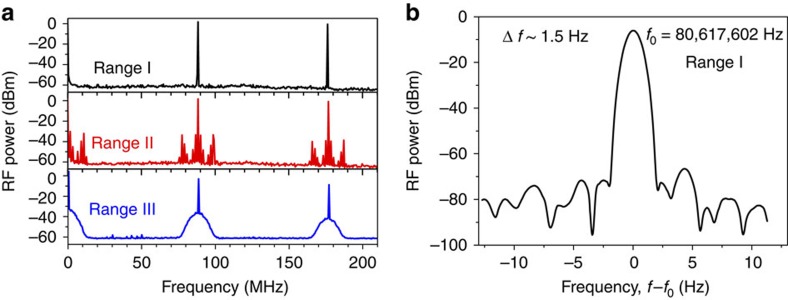
Radio frequency intermode beat spectra. (**a**) Typical radio frequency (RF) spectra for ranges I, II and III of the modulation frequencies presented in Figs 2d and 3a taken with the resolution of 1 kHz. (**b**) High-resolution (with the resolution of 1 Hz) spectrum with full-width half maximum Δ*f*∼1.5 Hz for narrow peak emission (range I in Fig. 2d) at modulation frequency near 80.61 MHz.

To assess the phase stability of the emission pulses, radio frequency intermode beat spectra of their intensity are measured with an electronic spectrum analyser. Three distinctively different types of the radio frequency spectra have been observed (Fig. 4a). For the modulation near and slightly below the resonance frequency or its higher harmonics (ranges I in both Figs 2c and 3a) the intermode beat spectra exhibit sharp peaks at the modulation frequency and its harmonics, rising straight from the noise level. Their FWHM is estimated to be <1.5 Hz (Fig. 4b) and is likely to be limited by the resolution of the spectrum analyser and the stability of the radio frequency master generator. This means that the emission pulses in the narrow peak laser spectra region are indeed mode-locked and the relative phases of the modes remain stable for at least 10^8^ pulses. For larger detuning from the resonant frequencies (ranges III in both Figs 2c and 3a), the radio frequency spectra have developed a ∼20-MHz-wide pedestal (Fig. 4a), indicating much reduced pulse amplitude stability and phase coherence. Radio frequency spectra for the modulation conditions in ranges II in both Figs 2c and 3a are typically even more complex and broad (Fig. 4a), and usually consist of several additional peaks near the modulation frequency and its harmonics corresponding to an amplitude modulation, often accompanied by the broadening of the main central peak.

## Discussion

**Figure 5:**
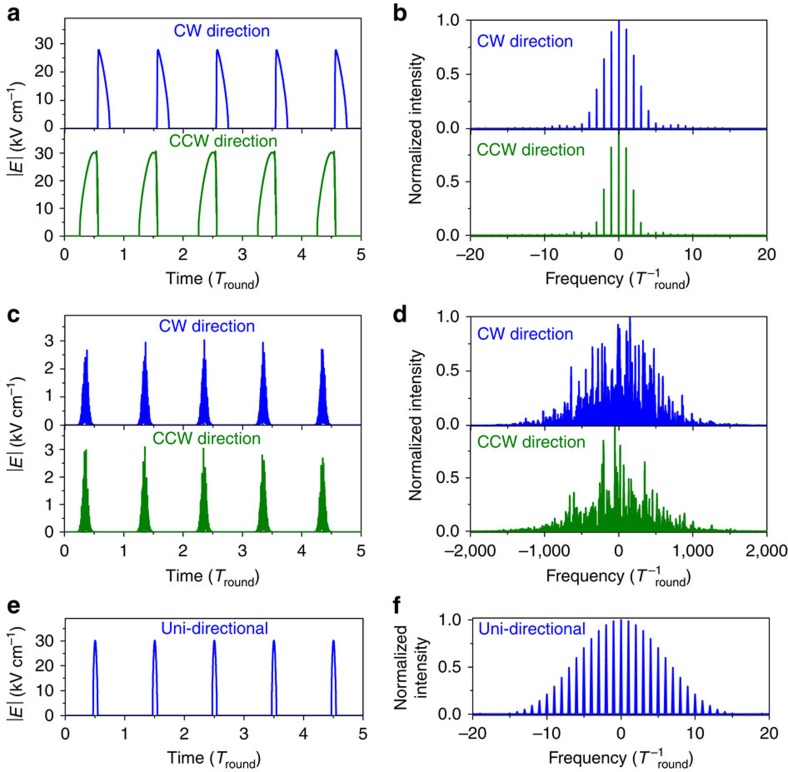
Modelling of external ring cavity QCL. Calculated laser output under resonant modulation of the bias. The time and the frequency are normalized by the round-trip time *T*_round_ in an external cavity. (**a**) Absolute values of the CW and CCW-propagating electric fields *|E|* and (**b**) their spectra for a sinusoidal modulation of the bias with the modulation period equal to *T*_round_. (**c**) Absolute values of the electric fields and (**d**) their spectra for a sinusoidal modulation of bias with the modulation period equal to 1.01·*T*_round_. (**e**) Absolute value of the electric field and (**f**) its spectrum when the bias is a sequence of Gaussian pulses with the modulation period equal to *T*_round_ and duration of 0.2·*T*_round_.

We model AML in an external cavity QCL with space- and time-domain simulations using a four-subband model for the QCL active region (Methods section). Figure 5a,b shows examples of the absolute values of the CW and CCW-propagating laser fields and their spectra when the bias is sinusoidally modulated with the frequency exactly at the resonance with the round-trip frequency. The time and the frequency in all theoretical plots are normalized by the round-trip time *T*_round_ for an external-cavity length of 1.29 m. The direct current (DC) level of the bias is close to the threshold and the maximum amplitude of the current is equal to 1.77·*I*_th_. In this example the high-intensity pulses are present in both directions, which should lead to potentially strong SHB effects. However, the comparison of the CW and CCW pulses in Fig. 5a reveals an interesting feature: the pulses propagating in the opposite directions experience a self-consistent shift in their shape and relative phase of propagation which allows them to avoid an overlap in the gain medium. This mutual avoidance maximizes the gain available to each pulse and is entirely the result of their interaction through the coherent population grating, that is, the SHB effect. The tendency of laser emission to avoid an overlap in time, space or spectral domain to ‘feed' from unsaturated gain regions is of course a generic property of any laser. Since QCLs have a homogeneously broadened gain transition, the only mechanism which could facilitate interaction between the pulses is SHB. If the SHB is ‘turned off' by forcing the population grating amplitude to be equal to zero at all times, the pulses will have identical shape and completely overlap in the gain medium.

The pulse sides appear to be very sharp because the rise and fall times of the pulses are controlled by the gain recovery timescale which is three orders of magnitude shorter than the round-trip time.

Since the pulses do not overlap in the gain medium, there is no mode proliferation with random phases and the pulses are essentially Fourier-transform limited, with narrow spectra as shown in Fig. 5b. The pulse shapes may vary from run to run due to the presence of fluctuations and spontaneous emission noise in our model, but this avoidance feature is present whenever lasing occurs in both propagating directions. In most runs, lasing in one direction eventually dies out over a long time scale of many hundreds or thousands of round trips. In the experiment the two-directional lasing regime with narrow spectra is more stable.

The above physical picture of the pulse mutual avoidance and the resulting narrow spectra exists exclusively in the close vicinity of the resonance between the modulation period and the round-trip time. When the detuning is introduced, the pulses are not able to avoid each other in the active medium. Their mutual scattering on the coherent population grating leads to proliferation of longitudinal modes with uncorrelated phases. In this case SHB has a detrimental effect, similarly to AML in monolithic QCLs. The spectra experience a dramatic broadening to many thousands of external-cavity modes and the pulses develop a substructure in time domain, with ultrafast oscillations on the picosecond timescale. This regime is illustrated in Fig. 5c,d for the case of modulation frequency 1% lower compared with the round-trip frequency and the modulation amplitude corresponding to the maximum current amplitude of 1.15·*I*_th_. With further detuning in any direction the qualitative pattern remains the same but the pulse amplitude decreases until the laser ceases to operate.

The theoretical findings of the pulses with the narrow spectra in the vicinity of the resonance followed by the dramatic expansion of the spectra and eventual shutdown of the laser with modulation frequency detuning are in good qualitative agreement with the experimental data. The modelling also shows that one can achieve much shorter pulses and broader phase-locked frequency combs by modulating the pumping with shorter and sharper pulses instead of the sinusoidal modulation. Figure 5e,f presents the laser output electric field and the corresponding spectrum when the bias is a sequence of Gaussian pulses with zero DC offset and 0.2·*T*_round_ duration at the *e*^−1^ level. The pulse repetition rate is exactly equal to the round-trip frequency and the peak current amplitude is 1.77·*I*_th_. In this case of narrower pumping pulses, the uni-directional regime is achieved much faster. The resulting pulses, which propagate only in one direction, are not affected by the SHB effects; they have a nice symmetric shape and Fourier-transform limited spectra. The surviving lasing direction fluctuates from CW to CCW randomly from run to run. For practical purposes one can introduce an optical isolator into the cavity to select one definite lasing direction.

When the modulation period is slightly detuned from resonance, the lasing fields in both directions can survive. Similar to the case of sinusoidal modulation, pulses interact strongly in the laser chip due to scattering on the coherent population grating, resulting in the ultrafast substructure and much broader spectra. This regime shows long-term instabilities: the pulses demonstrate amplitude modulation which fluctuates from run to run, and in many cases the lasing dies out in one direction. Again, the use of an optical isolator would be beneficial.

With decreasing duration of the driving pulses, the output laser pulses become shorter. The simulations for the varying width of the voltage pulses have revealed that the shortest output pulse duration is limited, in order of magnitude, by the time it takes the pulse to travel along the laser chip, which is of the order of 10–45 ps for a 1–4.5-mm chip. Much shorter pulses will experience gain only in a small part of the laser chip. The corresponding voltage pulses will have the duration of ∼30–60 ps, which is at the limit of capabilities of modern electronics.

Since SHB does not destroy mode locking in the presence of counter-propagating pulses, one should expect that the AML is also possible in a Fabry–Perot cavity configuration. Indeed the simulations done for a Fabry–Perot cavity yield the results qualitatively similar to those for a ring cavity. They will be reported elsewhere, together with experimental data.

In conclusion, robust, stable generation of actively mode-locked pulses has been demonstrated from a room temperature *λ*∼5 μm external-cavity QCL driven by the periodically modulated current with the modulation frequency slightly below the round-trip cavity frequency of 80.7 MHz or its harmonics. The AML has been achieved at driving current amplitudes covering the entire dynamic range of the laser. An ultra-narrow radio-frequency intermode beat linewidth below 1.5 Hz was recorded. Introduction of a diffraction grating into the ring cavity provides a single-peak emission tuning range of ∼150 cm^−1^. The theoretical simulations are in good agreement with the observed dynamics and shed light on the physical mechanism of AML in QCLs.

## Methods

### Experimental set-up

The free-space X-shaped external ring cavity QCL set-up (Fig. 1) is very similar to one described in ref. 15 and includes ‘Alpes Lasers' QCL, two anti-reflection coated 4 mm focal length chalcogenide lenses, four flat uncoated silver mirrors and a 0.5-mm-thick CaF_2_ beam-splitter anti-reflection coated on one side to suppress Fabry–Perot interference in it and to provide ∼5% reflectivity to out-couple the light. The temperature of the laser chip submount is kept at 15 °C. The length of the cavity of 3.4–3.8 m has been chosen to clearly resolve the periodic emission pulses expected at the fundamental round-trip time (∼11–12 ns) and at its higher harmonics, by a stand-alone ‘Vigo' PVI-2TE-6 mercury cadmium telluride detector, which has a rise time of ∼2 ns. A Fourier-transform infrared ‘Bruker IFS66' spectrometer was utilized for spectral recordings and a thermopile detector was used for average optical power measurements. An ‘Avtech' AV-1011-C pulse generator providing 1 μs−1 ms-long pulses with up to a 50% duty cycle was used to align the ring cavity set-up prior the resonant modulation experiments. The laser threshold current in the pulsed non-resonant regime is ∼400 mA with the roll-over current at ∼750 mA. The remaining reflectivity of <2% of the anti-reflection coated laser chip facets reduces the Fabry–Perot feedback from these facets to such an extent that the laser no longer operates as a free-running laser device outside the external cavity. The ring cavity QCL was also tested in the continuous wave regime demonstrating performance characteristics similar to those reported in ref. 15. The sinusoidal electrical signal with variable frequency and amplitude is supplied by an ‘Agilent' 8648C radio frequency master generator. The output from the generator is connected to a specially designed interface board acting as a broadband (∼60–400 MHz) power amplifier with adjustable amplification based on a high-frequency high-power LDMOS FET transistor. The interface board is designed to provide negative current of up to ∼1 A on 50 Ω impedance load, to have zero DC bias offset and strong rejection of the signal with the positive polarity. To monitor the shape and the amplitude of the driving current a ∼3-ns rise time inductive current probe has been introduced directly before the QCL chip. The outputs from the current probe and the mercury cadmium telluride detector are connected to an oscilloscope with 300 MHz bandwidth. Because of the interface board design and the voltage-dependent resistance of the QCL, the resulting shape of the current modulation swinging from zero value to the operating values is found to be not perfectly sinusoidal (Fig. 2a). Due to the high-impedance mismatch with the QCL, which results in the strong reflected radio frequency signal, the shape of the driving current observed on the oscilloscope does not necessarily represent the current applied directly to the QCL chip. For that reason the exact measurements of the current values are unreliable and all current amplitudes are therefore given only as multiples of the threshold current.

### Theoretical modelling

The theoretical modelling of AML in an external-cavity QCL includes full spatiotemporal dynamics of the laser field, intersubband polarization and electron populations within coupled Maxwell and density-matrix equations. The active region model is described in detail in ref. 9. We use a comprehensive model of the QCL active region and transport that includes resonant tunnelling injection, electron distribution over in-plane *k*-vectors and space charge. We also perform time-domain and space-domain simulations of the propagating field, in which we can directly follow the pulse formation in time. Note that previous studies such as ref. 13 and ref. 14 used the frequency-domain modal approach and a two- or three-level laser model without current injection. We modified the field propagation model as compared with ref. 9 to include appropriate geometry and boundary conditions for an external cavity. Ideal anti-reflection coatings on both facets of the laser and 5% out-coupling from the external cavity for both directions are assumed for all presented simulations. It was found that additional residual facet reflectivities of 1% have negligible effect on the results. A shorter external cavity of 1.29 m was used to reduce calculation times. The results normalized by the cavity length and the round-trip time are not sensitive to the increase in the cavity length to 3.8 m. The input parameter in the model is the applied bias voltage and not the current, which is calculated self-consistently taking into account electron distributions in the subbands. Therefore the current response to a sinusoidal modulation of the bias is non-sinusoidal, but the nonlinearity in the response is small for the used modulation amplitudes.

## Additional information

**How to cite this article:** Revin, D. G. *et al*. Active mode locking of quantum cascade lasers in an external ring cavity. *Nat. Commun.* 7:11440 doi: 10.1038/ncomms11440 (2016).
